# KIF1Bβ increases ROS to mediate apoptosis and reinforces its protein expression through O_**2**_^**−**^ in a positive feedback mechanism in neuroblastoma

**DOI:** 10.1038/s41598-017-17192-6

**Published:** 2017-12-04

**Authors:** Clara Angelina, Irene Sze Ying Tan, Zhang’e Choo, Oswald Zhao Jian Lee, Shazib Pervaiz, Zhi Xiong Chen

**Affiliations:** 1 Department of Physiology, Yong Loo Lin School of Medicine, National University of Singapore, Singapore, Singapore; 2 NUS Graduate School for Integrative Sciences and Engineering, National University of Singapore, Singapore, Singapore; 3 Singapore-MIT Alliance, Singapore, Singapore; 4 KK Women’s and Children’s Hospital, Singapore, Singapore

## Abstract

Relapse-prone, poor prognosis neuroblastoma is frequently characterized by deletion of chr1p36 where tumor suppressor gene *KIF1Bβ* resides. Interestingly, many 1p36-positive patients failed to express KIF1Bβ protein. Since altered cellular redox status has been reported to be involved in cell death and protein modification, we investigated the relationship between reactive oxygen species (ROS) and KIF1Bβ. Here, we showed that wild-type KIF1Bβ protein expression positively correlates with superoxide (O_2_^−^) and total ROS levels in neuroblastoma cells, unlike apoptotic loss-of-function KIF1Bβ mutants. Overexpression of KIF1Bβ apoptotic domain variants increases total ROS and, specifically O_2_^−^, whereas knockdown of endogenous KIF1Bβ decreases ROS and O_2_^−^. Interestingly, O_2_^−^ increases KIF1Bβ protein expression, independent of the proteasomal degradation pathway. Scavenging O_2_^−^ or ROS decreases KIF1Bβ protein expression and subsequent apoptosis. Moreover, treatment with investigational redox compound Gliotoxin increases O_2_^−^, KIF1Bβ protein expression, apoptosis and colony formation inhibition. Overall, our findings suggest that ROS and O_2_^−^ may be important downstream effectors of KIF1Bβ-mediated apoptosis. Subsequently, O_2_^−^ produced may increase KIF1Bβ protein expression in a positive feedback mechanism. Therefore, ROS and, specifically O_2_^−^, may be critical regulators of KIF1Bβ-mediated apoptosis and its protein expression in neuroblastoma.

## Introduction

Neuroblastoma is the most common childhood extracranial solid tumor accounting for 10% of all pediatric cancers in the United States and 5.3% of all pediatric cancers in Singapore^1–3^. Due to its complexity, neuroblastoma can undergo regression and differentiation to become benign ganglioneuroma or oncogenic transformation into unfavorable metastatic tumors during diagnosis. High-risk neuroblastoma has been associated with chromosome 1p36 deletion^4^. Chromosome 1p36 contains a bonafide tumor suppressor gene called KIF1Bβ which is likely to be involved and defective in various cancers including neuroblastoma^5^.

KIF1Bβ is a haploinsufficient 1p36 tumor suppressor and a downstream target of EglN3, which was found necessary and sufficient to mediate Nerve Growth Factor (NGF) withdrawal-induced apoptosis during neural crest development^6,7^. Neuroblasts which are out-competed for NGF during sympathetic nervous system development upregulate EglN3 to induce KIF1Bβ, promote RNA Helicase A (DHX9) nuclear translocation and lead to apoptosis through increased XAF1 expression. Failure of developmental apoptosis due to loss of KIF1Bβ may lead to improper survival of neuroblasts during NGF signaling phase of sympathetic nervous system development, predisposing tumorigenesis^6–8^. Previously, we have also showed that KIF1Bβ protein expression does not always faithfully recapitulate patients’ 1p36 genotype. One out of two 1p36^+/+^ and majority of 1p36^+/−^ neuroblastoma patients failed to express KIF1Bβ protein^6^. Furthermore, given that EglN3-mediated increase in KIF1Bβ protein expression is independent of transcription, the intermediate responsible for altering KIF1Bβ protein expression remains unknown^7^.

Separately, reactive oxygen species (ROS) has been well-studied for its role in various signaling pathways that are involved in key biological functions such as cell proliferation, differentiation and cell death^9^. Excessive ROS can induce oxidative stress, causing irreversible cell damage and cell death. On the other hand, slight increase of specific species may transiently promote biological changes involved in cell growth and differentiation. Thus, the biphasic and species-specific properties of ROS is important in determining cell fate^10,11^. Altered cellular redox status is known to be implicated in tumorigenesis. Cancer cells often exhibit high metabolic activity that requires increased ATP to maintain biological processes such as uncontrolled proliferation. Increased ATP production is accompanied by increased ROS generation, leading to oxidative stress observed in most cancers. In order to cope with the oxidative burden, cancer cells increase their ROS-tolerating threshold by enhancing the expression of antioxidant enzyme^9,10^. Furthermore, ROS-mediated genetic alterations and protein modifications may confer a survival advantage by creating a permissive environment for cancer cells to tolerate high ROS exposure. ROS also serves as secondary messengers in signaling cascades whereby it appears to be involved in transcriptional activation and other receptor-mediated signaling pathways^9,12,13^.

Therefore, taking into consideration the multiple roles of ROS including cell death and protein modification, we investigated the relationship between KIF1Bβ and ROS. Specifically, given the recent role of KIF1Bβ in mitochondrial dynamics and apoptosis, we asked if KIF1Bβ could lead to ROS production and subsequent cell death^14^. Furthermore, we asked if ROS could be the missing intermediate in regulating KIF1Bβ protein expression and thereby resolving the enigma of absent KIF1Bβ protein expression in 1p36^+/−^ and 1p36 ^+/+^ neuroblastoma patients.

## Results

### Endogenous total ROS and O_2_^−^ levels correspond to KIF1Bβ expression in neuroblastoma cells

**Figure 1:**
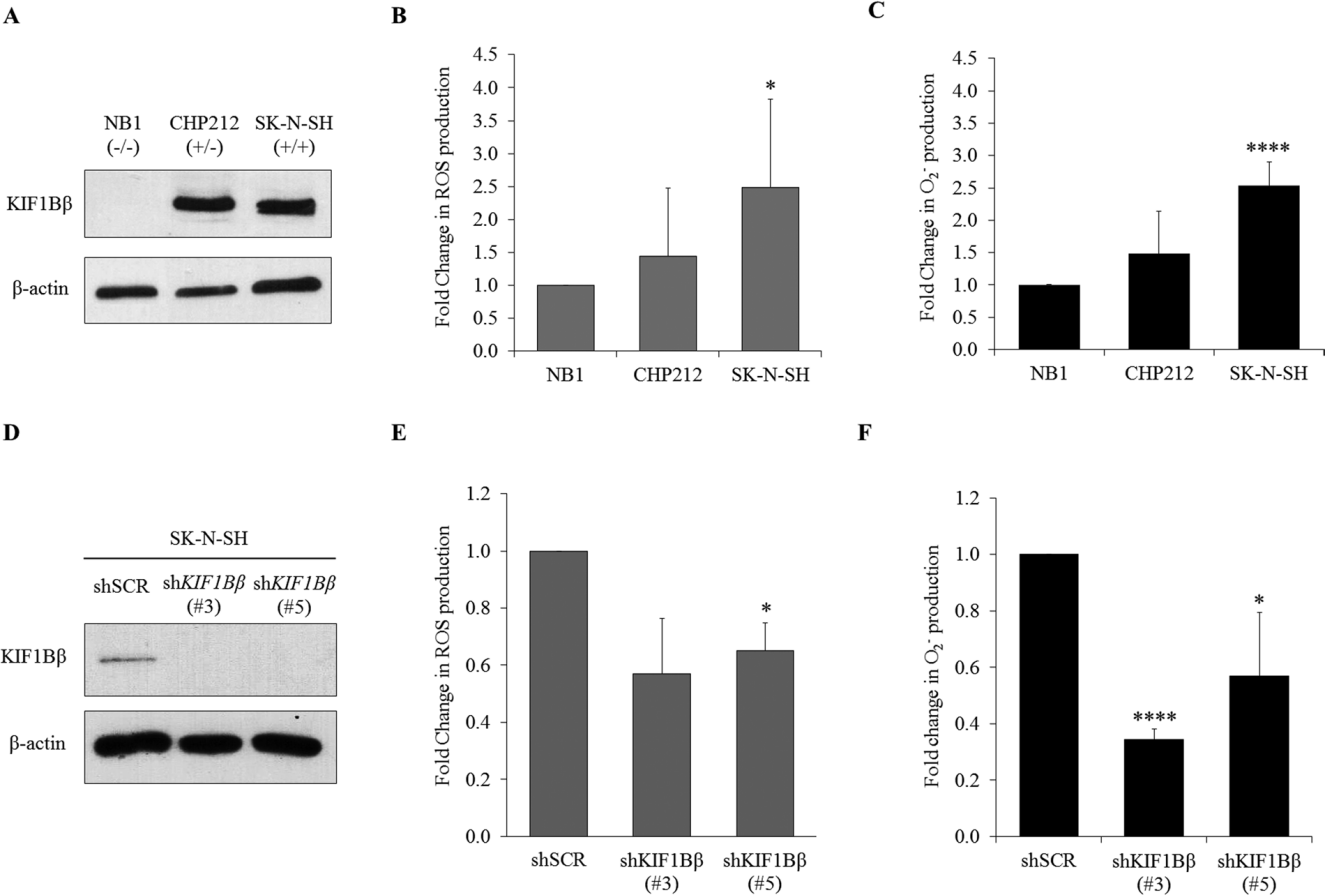
Endogenous total ROS and O_2_^−^ levels correspond to KIF1Bβ expression in neuroblastoma cells. **(A)** Immunoblot analysis of KIF1Bβ in neuroblastoma cell lines NB1, CHP212 and SK-N-SH. **(B)** Flow cytometric analysis of intracellular ROS with DCFDA (mean ± SD; n = 3; *P < 0.05). **(C)** Intracellular O_2_^−^ measured with lucigenin-based chemiluminescence assay (mean ± SD; n = 3; ****P < 0.0001). **(D)** Immunoblot analysis of SK-N-SH cells stably transduced with lentivirus encoding shRNAs targeting KIF1Bβ (sh*KIF1Bβ* #3 and #5) or control virus (shSCR) and selected with 1 µg/ml puromycin for 8 days. **(E**) Corresponding fold change in flow cytometric analysis of intracellular ROS and **(F)** Fold change in intracellular O_2_^−^ determined using lucigenin-based chemiluminescence assay of SK-N-SH cells stably transduced with lentiviruses as indicated (mean ± SD; n = 3; *P < 0.05; ****P < 0.0001).

To determine the correlation between ROS and KIF1Bβ expression levels, endogenous total ROS and O_2_^−^ levels were measured across neuroblastoma cell lines with different KIF1Bβ genotypic profiles based on their 1p36 status (Fig. 1A). The cell lines are NB1 (1p36^−/−^), CHP212 (1p36^+/−^) and SK-N-SH (1p36^+/+^). KIF1Bβ-expressing cell line SK-N-SH has significantly higher ROS and O_2_^−^ levels compared to KIF1Bβ-null cell line NB1 (Fig. 1B and C). Indeed, silencing of KIF1Bβ using two independent shRNAs in SK-N-SH neuroblastoma cells resulted in decreased intracellular ROS and O_2_^−^ levels (Fig. 1D–F). Overall, the results suggest a pro-oxidant function of KIF1Bβ and a positive association between the expression of KIF1Bβ and the levels of total ROS and O_2_^−^.

### Apoptotic function of KIF1Bβ is necessary for ROS induction in neuroblastoma cells

**Figure 2:**
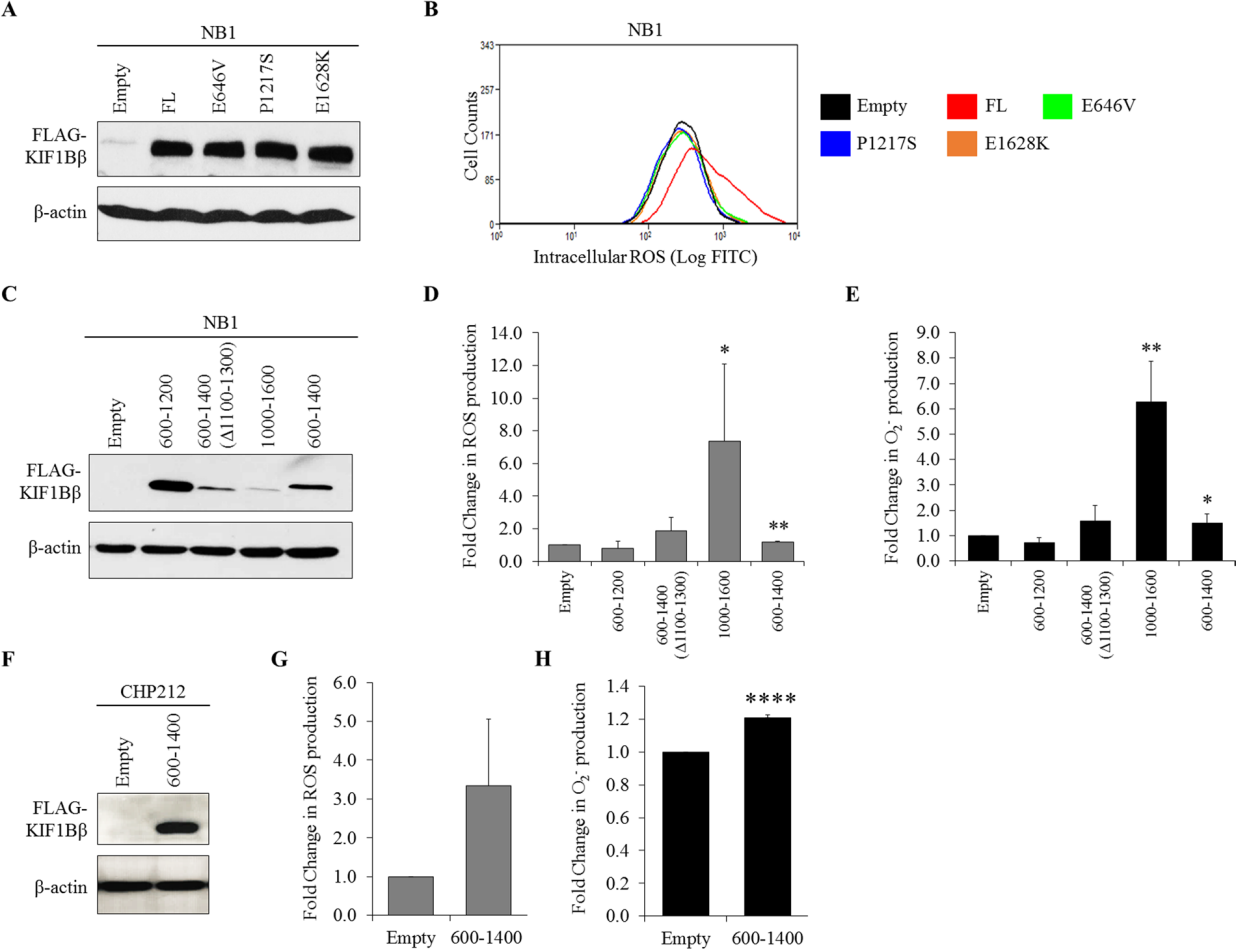
Apoptotic function of KIF1Bβ is necessary for ROS induction in neuroblastoma cells. **(A)** Immunoblot analysis of NB1 cells after 24 hours of transient transfection with 4 µg wild-type (FL) FLAG-KIF1Bβ or FLAG-KIF1Bβ mutants (E646V, P1217S, E1628K). Empty vector pcDNA3 (Empty) served as negative control. **(B)** Corresponding flow cytometric analysis of intracellular ROS in NB1 cells 24 hours post-transfection with FLAG-KIF1Bβ mutants as indicated. **(C)** Immunoblot analysis of NB1 cells after 24 hours of transient transfection with empty vector pcDNA3 (Empty) or FLAG-KIF1Bβ domain variants as indicated. **(D)** Corresponding fold change in flow cytometric analysis of intracellular ROS and **(E)** Fold change in intracellular O_2_^−^ determined using lucigenin-based chemiluminescence assay 24 hours post-transfection in NB1 cells (mean ± SD; n = 3; *P < 0.05; **P < 0.01). **(F)** Immunoblot analysis of CHP212 cells after 24 hours of transient transfection with empty vector pcDNA3 (Empty) or FLAG-KIF1Bβ600-1400. **(G)** Corresponding fold change in flow cytometric analysis of intracellular ROS and **(H)** Fold change in intracellular O_2_^−^ determined using lucigenin-based chemiluminescence assay 24 hours post-transfection in CHP212 cells (mean ± SD; n = 3; ****P < 0.0001).

After determining the pro-oxidant function of KIF1Bβ, we next investigate whether ROS is involved in KIF1Bβ-mediated apoptosis in neuroblastoma. KIF1Bβ mutants previously identified in neuroblastoma and pheochromocytoma patients with loss-of-function in apoptosis were tested (Fig. 2A)^6^. Ectopic expression of wild-type KIF1Bβ markedly increased intracellular ROS production compared to empty vector control in NB1 cells whereas the overexpression of disease-causing apoptotic loss-of-function KIF1Bβ mutants, E646V, P1217S and E1628K, failed to induce intracellular ROS production (Fig. 2B). Moreover, additional testing using previously characterized apoptotic and non-apoptotic domains KIF1Bβ variants showed that ectopically expressed apoptotic KIF1Bβ variants, KIF1Bβ1000-1600 and KIF1Bβ600–1400, significantly increased the production of intracellular ROS and O_2_^−^ in NB1 cells (Fig. 2C–E)^6^. Non-apoptotic KIF1Bβ variants, KIF1Bβ600–1200, on the other hand, have no effect on intracellular ROS and O_2_^−^ levels whereas KIF1Bβ600–1400(Δ1100–1300), showed modest increase that was not statistically significant (Fig. 2C–E). Furthermore, exogenously expressed apoptotic variant KIF1Bβ600–1400 in CHP212 cells also caused the induction of intracellular ROS and O_2_^−^ (Fig. 2F–H). Interestingly, ectopic introduction of apoptotic variant KIF1Bβ600–1400 and wild-type KIF1Bβ have no effect on mitochondrial O_2_^−^ levels in NB1 cells, demonstrating the site-specific regulation of KIF1Bβ on ROS (Fig. S1A and B). Taken together, these results demonstrate that endogenous total ROS and O_2_^−^ induction is specific to and dependent on KIF1Bβ apoptotic function.

### KIF1Bβ requires ROS, specifically O_2_^−^, to induce apoptosis in neuroblastoma cells

**Figure 3:**
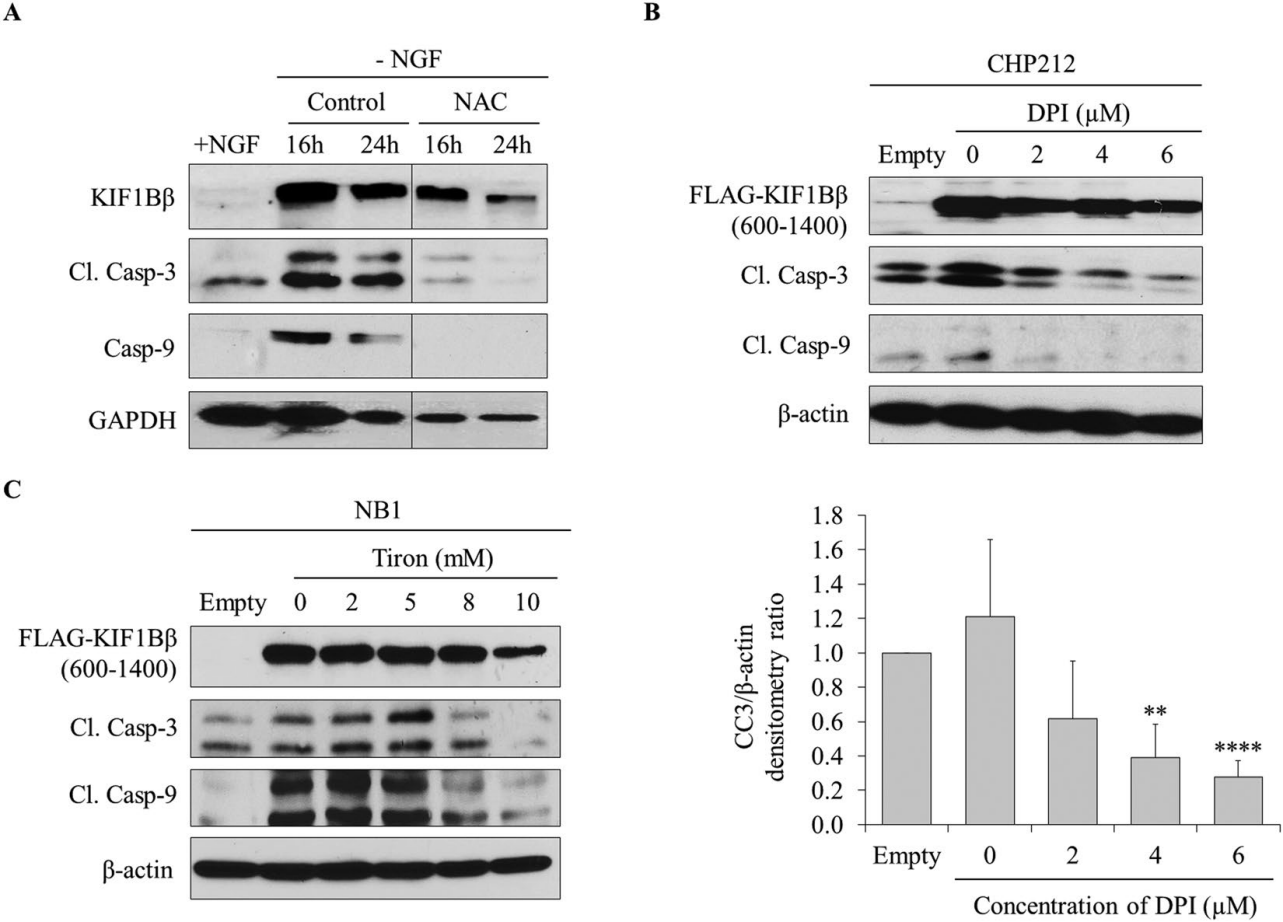
KIF1Bβ requires ROS, specifically O_2_^−^, to induce apoptosis in neuroblastoma cells. **(A)** Immunoblot analysis of differentiated PC12 cells with (+) and without (−) NGF as indicated. Differentiated PC12 cells were treated with or without (control) NAC prior to NGF withdrawal and replenished every 12 hours after NGF withdrawal for treated samples. **(B)** Immunoblot analysis of CHP212 cells transfected with 4 µg FLAG-KIF1Bβ600–1400 for 24 hours followed by increasing doses of DPI treatment for 12 hours. Bottom – corresponding densitometry for cleaved caspase-3 **(**CC3) expression (mean ± SD; n = 3; **P < 0.01; ****P < 0.0001). **(C)** Immunoblot analysis of NB1 cells transfected with 4 µg FLAG-KIF1Bβ600–1400 for 24 hours followed by increasing doses of Tiron treatment for 6 hours.

Motivated by our findings that KIF1Bβ, in the presence of its apoptotic activity, regulates the production of ROS and O_2_^−^, we investigated whether ROS is required for KIF1Bβ to mediate apoptosis in neuroblastoma or whether ROS production is a consequence of KIF1Bβ-induced apoptosis. To do so, first we used differentiated PC12 cells to study the involvement of ROS during neuronal survival by NGF. Consistent with previous reports, NGF withdrawal from differentiated PC12 cells resulted in the induction of KIF1Bβ protein expression, and increased apoptosis as determined by cleaved caspase-3 (Fig. 3A)^7^. Interestingly, caspase-9 protein expression was also induced (Fig. 3A). Importantly, we observed that treatment of differentiated PC12 cells with ROS scavenger N-acetylcysteine (NAC) protected PC12 cells from apoptosis by abolishing cleaved caspase-3 and induction of caspase-9 despite the presence of KIF1Bβ expression upon NGF withdrawal, suggesting that ROS is a downstream effector of KIF1Bβ and is necessary for NGF withdrawal-dependent apoptosis (Fig. 3A).

As our earlier finding showed that silencing KIF1Bβ protein expression resulted in greater reduction of O_2_^−^ than total ROS, we asked if blocking O_2_^−^ production would be sufficient in rescuing neuroblastoma cells from KIF1Bβ-mediated apoptosis (Fig. 1D–F). Indeed, protection from apoptosis induced by ectopic expression of apoptotic variant KIF1Bβ600-1400 was observed in CHP212 cells treated with DPI, an inhibitor that blocks NADPH oxidase production of O_2_^−^ (Fig. 3B). Similarly, treatment of NB1 cells with Tiron, a scavenger for NADPH oxidase-produced O_2_^−^, protected the cells from KIF1Bβ-mediated apoptosis (Fig. 3C). Together, these results suggest that ROS, specifically O_2_^−^, is required for KIF1Bβ to induce apoptosis in neuroblastoma.

### O_2_^−^ increases KIF1Bβ protein expression in a positive feedback loop

**Figure 4:**
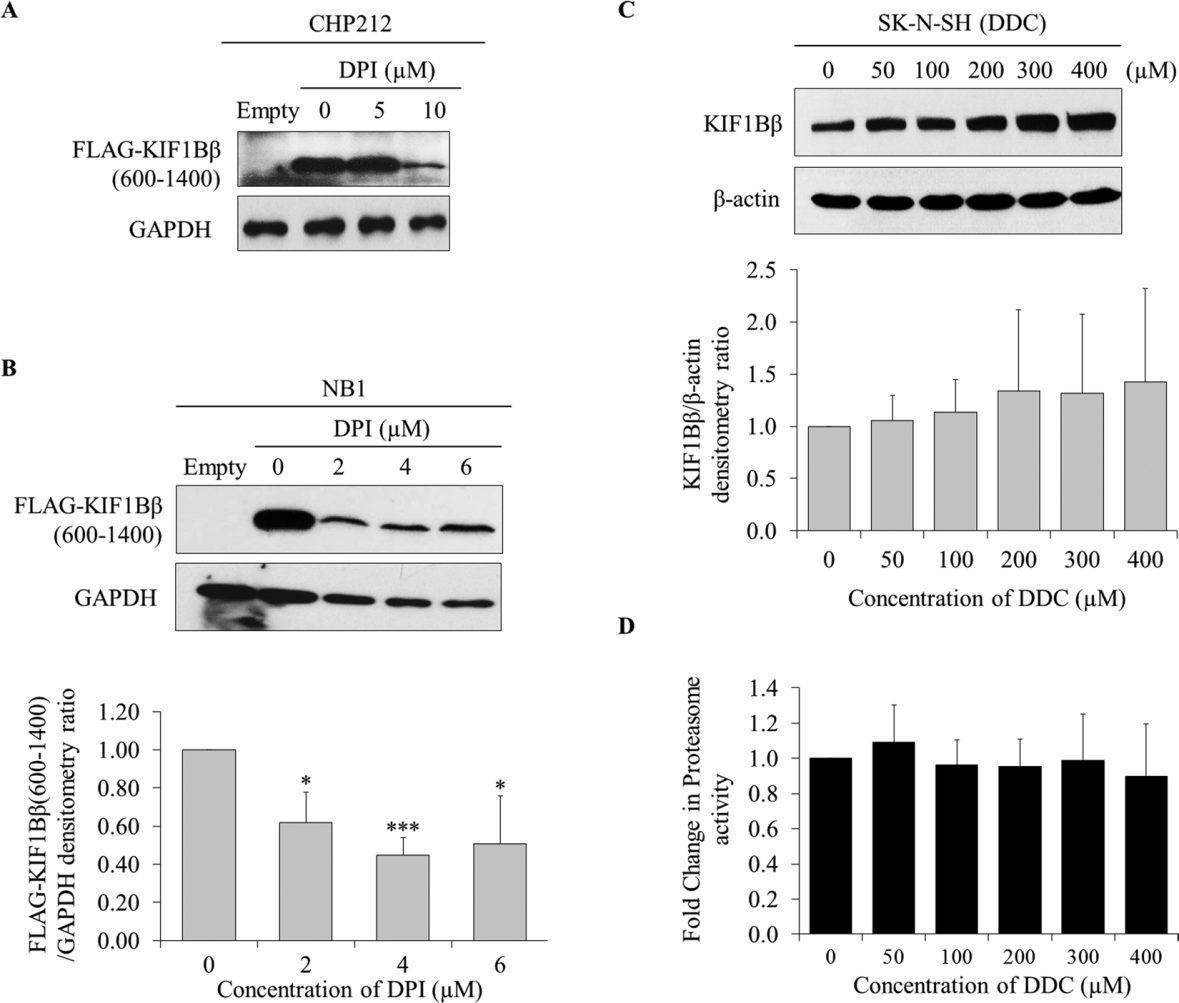
O_2_^−^ increases KIF1Bβ protein expression in a positive feedback loop. **(A)** Immunoblot analysis of CHP212 cells transfected with 4 µg empty vector pcDNA3 (Empty) or FLAG-KIF1Bβ600–1400 for 24 hours followed by increasing doses of DPI treatment for 12 hours. **(B)** Immunoblot analysis of NB1 cells transfected with 4 µg empty vector pcDNA3 (Empty) or FLAG-KIF1Bβ600–1400 for 24 hours followed by increasing doses of DPI treatment for 12 hours. Bottom – corresponding densitometry for FLAG-KIF1Bβ600–1400 expression (mean ± SD; n = 3; *P < 0.5; ***P < 0.001). **(C)** Immunoblot analysis of KIF1Bβ expression in SK-N-SH cells in response to increasing doses of DDC treatment for 4 hours. Bottom – corresponding densitometry for KIF1Bβ expression (mean ± SD; n = 3). **(D)** Fold change in proteasomal activity with increasing doses of DDC treatment.

Intriguingly, a reduction in KIF1Bβ protein level was observed in both NAC-treated differentiated PC12 cells upon NGF withdrawal and Tiron-treated NB1 cells at the highest dose, suggesting a possible feedback mechanism of ROS and O_2_^−^ on KIF1Bβ expression (Fig. 3A and C). To further validate this observation, we treated CHP212 and NB1 cells with NAC to remove total ROS and observed a concomitant reduction in exogenously expressed wild-type KIF1Bβ protein levels (Fig. S2A and B). Moreover, treatment of CHP212 cells at high dose (10μM) and NB1 cells with increasing doses of DPI to remove NADPH oxidase-produced O_2_^−^ resulted in an extensive reduction in ectopic expression of KIF1Bβ600–1400 protein levels, indicating O_2_^−^ may be a more specific and effective regulator of KIF1Bβ expression (Fig. 4A and B). Conversely, treatment of SK-N-SH cells with DDC, a SOD1 inhibitor, increased O_2_^−^ levels and resulted in a corresponding increase in endogenous KIF1Bβ protein levels (Figs 4C, and S2C,D). Together, these results suggest that KIF1Bβ increases ROS, specifically O_2_^−^, which is required for apoptosis and reinforcement of KIF1Bβ protein expression in a positive feedback loop in neuroblastoma cells.

Since ROS has been shown to modulate protein stability and turnover by interfering with the proteasomal degradation pathway, we asked the question if O_2_^−^ could also modulate KIF1Bβ protein expression via this mechanism^10^. However, there was no change in the proteasomal activity of SK-N-SH cells despite a dose-dependent increase in KIF1Bβ protein expression upon treatment with increasing doses of DDC (Figs 4C,D and S2E). This suggests that the positive regulation of O_2_^−^ on KIF1Bβ expression is independent of the proteasomal degradation pathway.

### Gliotoxin induces O_2_^−^ production to increase KIF1Bβ expression and apoptosis in neuroblastoma cells

**Figure 5:**
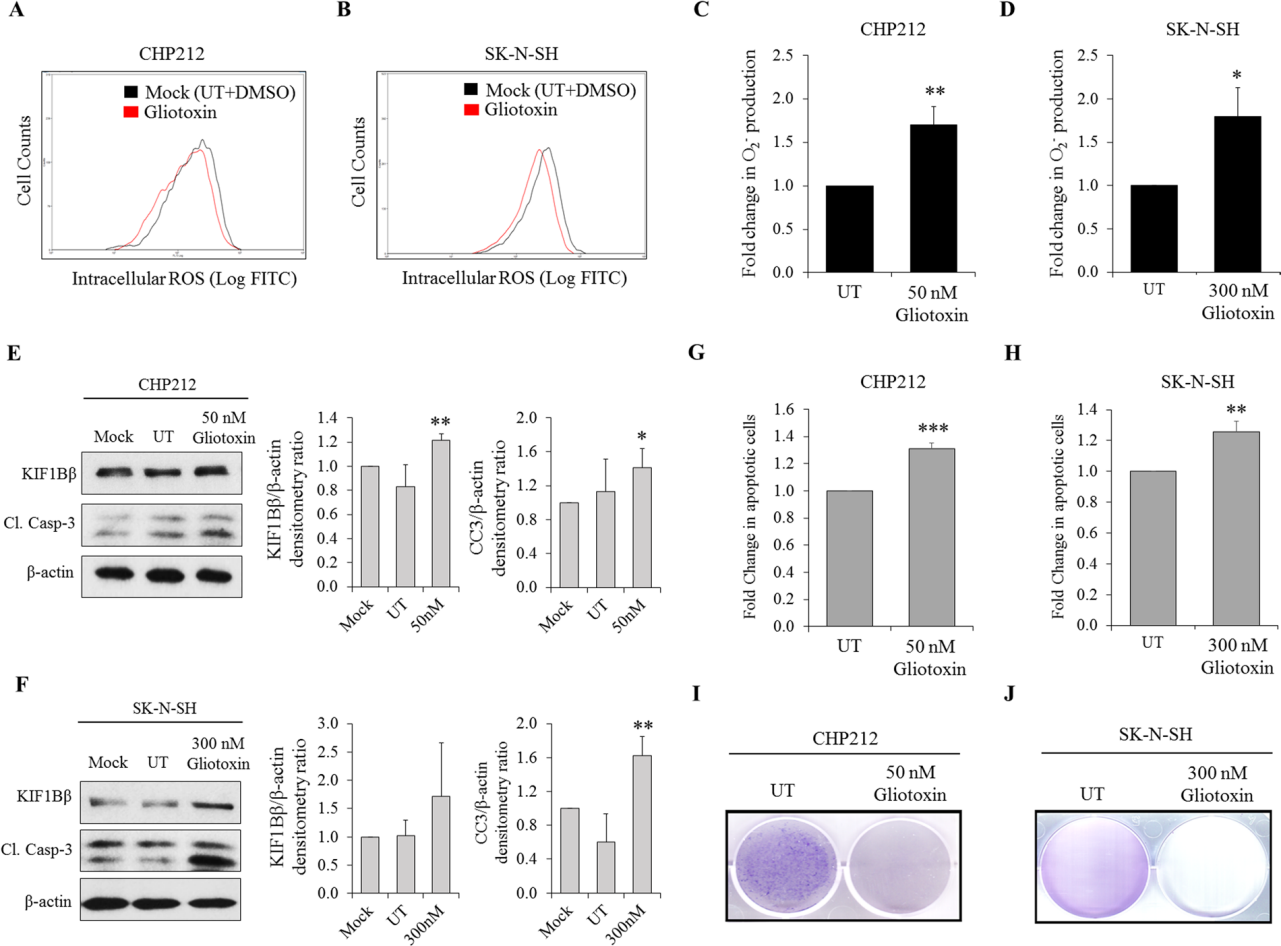
Gliotoxin induces O_2_^−^ to increase KIF1Bβ protein expression and apoptosis in neuroblastoma cells. **(A)** Flow cytometric analysis of intracellular ROS in CHP212 and **(B)** SK-N-SH cells after 1 hour of Gliotoxin treatment at 50 nM and 300 nM respectively. **(C)** Fold change in intracellular O_2_^−^ determined using lucigenin-based chemiluminescence assay in CHP212 and **(D)** SK-N-SH cells after 24 hours (50 nM) and 12 hours (300 nM) of Gliotoxin treatment respectively (mean ± SD; n = 3; *P < 0.05; **P < 0.01). **(E)** Immunoblot analysis of CHP212 and **(F**) SK-N-SH cells after 24 hours (50 nM) and 12 hours (300 nM) of Gliotoxin treatment respectively. Right – corresponding densitometry for KIF1Bβ and cleaved caspase-3 (CC3) expression (mean ± SD; n = 3; *P < 0.05; **P < 0.01). **(G)** Corresponding flow cytometric analysis of Propidium iodide/Annexin V (PI/AV) apoptotic cell staining in CHP212 and **(H)** SK-N-SH cells after 24 hours (50 nM) and 12 hours (300 nM) of Gliotoxin treatment respectively (mean ± SD; n = 3; **P < 0.01; ***P < 0.001). **(I)** Crystal violet staining to determine colony formation ability of CHP212 and **(J)** SK-N-SH cells that were treated with Gliotoxin for every 24 hours (50 nM) and 12 hours (300 nM) respectively, for several days.

**Figure 6:**
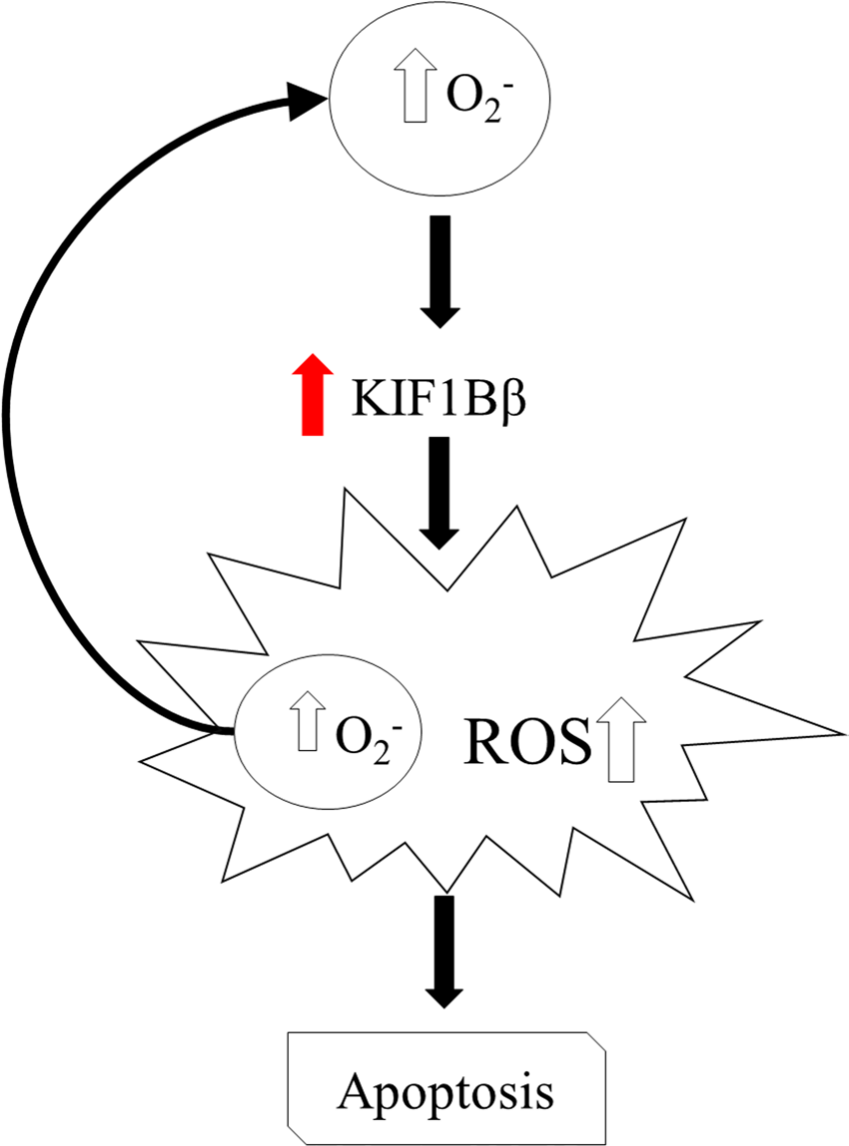
Proposed model of redox regulation of KIF1Bβ-mediated apoptosis in neuroblastoma cells. In neuroblastoma cells, KIF1Bβ protein expression induces the production of overall ROS, specifically O_2_^−^, which resulted in apoptosis. In turn, the increase in O_2_^−^ positively regulates the protein expression of KIF1Bβ, thus reinforcing KIF1Bβ-mediated apoptosis in neuroblastoma cells.

Since O_2_^−^ increases KIF1Bβ protein expression in neuroblastoma cells, we asked whether investigational redox compounds such as Gliotoxin can alter cellular redox status to increase O_2_^−^ level and corresponding KIF1Bβ protein expression to induce apoptosis in 1p36-intact neuroblastoma cells. To study whether Gliotoxin can modulate KIF1Bβ expression, CHP212 and SK-N-SH cells were treated with Gliotoxin in a dose-and time-dependent manner to determine optimal treatment dose and time for each cell line (Fig. S3A–D). Treatments of CHP212 (50 nM for 24 hours) and SK-N-SH cells (300 nM for 12 hours) with Gliotoxin increased O_2_^−^ level but had no effect on total ROS production (Fig. 5A–D). This corresponded to increased KIF1Bβ protein levels, cleaved caspase-3 expression, number of apoptotic cells, and decreased colony formation, suggesting that Gliotoxin may act through O_2_^−^ to increase KIF1Bβ protein expression to induce apoptosis (Figs 5E–J and S3E,F). Moreover, endogenous KIF1Bβ protein expression levels in Gliotoxin-treated CHP212 and SK-N-SH cells were reduced following Tiron treatment, thus indicating that O_2_^−^ is required for KIF1Bβ induction in Gliotoxin-treated cells (Fig. S3G and H). Taken together, we propose a model whereby O_2_^−^ and ROS may be critical regulators of KIF1Bβ-mediated apoptosis in neuroblastoma cells (Fig. 6).

## Discussion

KIF1Bβ has been reported as a potential tumour suppressor in neuroblastoma with respect to its crucial role in mediating developmental culling of NGF-deprived neuroblasts during NGF signalling phase of sympathetic nervous system development. Elevated ROS level has been shown to have a key role in the process of neuroblastoma cell apoptosis^15^. Our study suggests that there may be a relationship between ROS and KIF1Bβ protein expression and apoptotic function. Given the diverse roles of ROS in various signalling pathways, our findings reveal an additional function of ROS whereby it act as downstream effectors of KIF1Bβ-mediated apoptosis, while reinforcing KIF1Bβ protein expression through one of its species, O_2_^−^^16^.

Knowing that KIF1Bβ, total ROS and O_2_^−^ levels are positively correlated, we asked the question if KIF1Bβ could increase ROS to mediate apoptosis. Indeed, apoptotic loss-of-function KIF1Bβ mutants that were identified from patients, failed to upregulate total ROS compared to wild-type KIF1Bβ, suggesting that KIF1Bβ apoptotic function may indeed be dependent on increase in ROS. Our KIF1Bβ overexpression and silencing studies, and ROS scavenging experiments confirmed this and specifically, implicated O_2_^−^.

Intriguingly, O_2_^−^ was also able to increase KIF1Bβ protein expression with no change in proteasomal activity, suggesting that the direct or indirect effect of O_2_^−^ on KIF1Bβ is independent of the proteasome. It is interesting to note that ROS, specifically O_2_^−^, could be downstream and upstream of KIF1Bβ in a positive feedback loop. More importantly, although KIF1Bβ-mediated apoptosis could be rescued by NADPH oxidase inhibitor DPI and O_2_^−^ scavenger Tiron, and that KIF1Bβ protein expression is responsive to NADPH oxidase-produced O_2_^−^, overexpression of EglN3 was nevertheless shown to increase mitochondrial O_2_^−^ without affecting total ROS (Fig. S5A,C). Therefore, this suggests that during NGF signaling phase of sympathetic nervous system development from the neural crest, a possible mechanism may be for EglN3 to increase mitochondrial O_2_^−^ to upregulate KIF1Bβ protein expression. This is consistent with the function of KIF1Bβ in mitochondrial dynamics^14^. In turn, this leads KIF1Bβ to increase total ROS and NADPH oxidase-dependent O_2_^−^ for apoptotic induction and reinforcement of KIF1Bβ protein expression.

With our finding that O_2_^−^ may play a role in upregulating KIF1Bβ protein expression, we reasoned that investigational redox compounds such as Gliotoxin, a small molecule derived from fungal secondary metabolites, may potentially alter KIF1Bβ protein expression through the action of O_2_^−^. KIF1Bβ protein level was remarkably reduced in the presence of O_2_^−^ scavenger Tiron on Gliotoxin-treated cells, which highlights the important role of Gliotoxin-induced O_2_^−^ to upregulate KIF1Bβ in neuroblastoma cells. Therefore, treatment with Gliotoxin may potentiate KIF1Bβ-mediated apoptosis in neuroblastoma cells by increasing its protein expression through O_2_^−^.

Indeed, Gliotoxin treatment triggered early induction of O_2_^−^ without affecting total ROS, resulting in corresponding upregulation of KIF1Bβ protein expression, apoptosis and colony formation inhibition in CHP212 and SK-N-SH cells. This suggests that Gliotoxin-mediated increase in KIF1Bβ protein expression is likely due to upregulation of intracellular O_2_^−^, consistent with our earlier results. Additionally, Gliotoxin has been reported to act through thioredoxin redox system by accelerating NADPH oxidation and reducing intracellular H_2_O_2_, supporting the accumulation of O_2_^−^^17^. Taken together, our study suggests that 1p36-positive neuroblastoma patients who present with atypical, low or null KIF1Bβ protein expression may potentially benefit from Gliotoxin in order to re-express KIF1Bβ protein and mediate apoptosis in cancer cells.

## Methods

### Cell lines and cell culture

Human neuroblastoma cell lines (NB1, CHP212 and SK-N-SH) were maintained in RPMI-1640 (Hyclone) containing 10% fetal bovine serum (FBS; Hyclone), 1% 2.05 mM L-glutamine (Hyclone) and 1% penicillin-streptomycin. PC12 cells were maintained in DMEM containing 5% FBS (Hyclone), 10% horse serum (Hyclone) and 2% penicillin-streptomycin. All cells were obtained from American Type Culture Collection (ATCC) and cultured in a 37 °C, 5% CO_2_ humidified incubator.

### Transfection

Cells were transfected with 4 µg of plasmids containing either pcDNA3 or KIF1Bβ full-length/domain constructs/ variants using Lipofectamine^®^ 2000 Reagent (Invitrogen) in a ratio of 1:2.5 in Opti-MEM Reduced Serum Medium according to manufacturer’s protocol and incubated for 24 hours. Plasmids for wild-type KIF1Bβ, its domain constructs and mutants were generated previously^6,7^.

### shRNA lentiviral transduction

Lentiviruses encoding human KIF1Bβ shRNAs were made via co-transfection of shRNA-expressing lentiviral plasmids, packaging plasmids and pLP-VSVG into 293TN producer cell line^18,19^. The shRNA-expressing plasmids were gifts from Dr. Susanne Schlisio (Ludwig Institute for Cancer Research Ltd - Karolinska Institute, Stockholm, Sweden). Non-targeting virus (SCR) encoding shSCR plasmid was used as a negative control. The shRNA sequences targeting different coding regions of KIF1B gene were as follows:

shKIF1Bβ (#3) : 5′ - CTGGATTTGATGCGAGAGAT-3′

shKIF1Bβ (#5) : 5′ - GCCAAACTGGTTCGTGAATTA-3′

SK-N-SH cells were seeded 30% confluency in 60 mm culture dishes and volume of virus was added proportionally to the cellular confluency of cells. After 24 hours of transduction, cells were selected with medium containing 1 µg/ml of puromycin. Cells were selected for 8 days to achieve effective knockdown of KIF1Bβ, and they were subsequently harvested for immunoblot analysis and functional assays.

### Immunoblot analysis

Cells were harvested in EBC buffer (50 mM Tris at pH 8.0, 120 mM NaCl, 0.5% NP-40) containing protease inhibitors (Roche). Cell lysates were lysed and quantified by Bradford assay to obtain equal amount of protein extracts, then loaded and separated by SDS-polyacrylamide gel electrophoresis (SDS-PAGE). The proteins were then transferred onto a polyvinylidene difluoride (PDVF) membrane (Bio-Rad). Membrane blot were incubated with specific primary antibodies as follows: Rabbit polyclonal anti-KIF1Bβ was a gift from Dr. Susanne Schlisio. Mouse monoclonal anti-FLAG, rabbit monoclonal cleaved caspase-3, rabbit monoclonal PARP, rabbit polyclonal caspase-9 were purchased from Cell Signaling Technology. Mouse monoclonal β-actin and rabbit polyclonal GAPDH was purchased from Santa Cruz Biotechnology. Primary antibody signals were detected using either anti-rabbit or anti-mouse horseradish peroxidase-conjugated secondary antibodies (Cell Signaling Technology).

### Treatment with inhibitors and ROS scavengers

Stock solution of 10 mM Diphenyleneiodonium (DPI) (Sigma Aldrich), 1 M Sodium 4,5-dihydroxybenzene-1,3-disulfonate (Tiron) (Sigma Aldrich), 10mM N-acetylcysteine (NAC) (Sigma Aldrich) and 100 mM 5,10,15,20-Tetrakis(4-sulfonatophenyl)-21*H*,23*H*-porphyrin iron (III) chloride (FeTPPS) (Calbiochem) treatment was added to KIF1Bβ domain constructs-transfected cells or Gliotoxin-treated cells and then followed by Immunoblot analysis.

### Intracellular O_2_^−^ detection via lucigenin-based chemiluminescence assay

Cells were seeded at 80% confluency in a 60 mm culture dishes a day prior to drug treatment or transfection. Cells were harvested to measure intracellular O_2_^−^. Cell pellets were lysed with 450 µL of ATP releasing reagent (Sigma Aldrich). Stock solution of 10 mM lucigenin (N, N’-dimethyl-9,9′-biacridinium dinitrate; Sigma Aldrich) was automatically injected into the samples immediately upon cell lysis using Berthold Sirius Luminometer (Titertek-Berthold). Luminescence emitted was then detected and measured every 0.6-second over a period of 14.4 seconds.

### Flow cytometric analysis of intracellular ROS using DCFDA

Cells were seeded at 80% confluency in a 60 mm culture dishes a day prior to drug treatment or transfection. To measure intracellular ROS level, cell pellets were re-suspended in RPMI-1640 medium containing 5 µM of 5-(and-6)-chloromethyl-2-,7-dichlorofluorescin diacetate (CM-H2DCFDA; Molecular Probes, Life Technologies) and incubated in the dark at 37 °C for 20 minutes. Cell suspensions were further diluted and filtered for flow cytometric analysis using BD LSRFortessa^TM^ cell analyzer (BD Biosciences) with excitation and emission spectra of 495 nm and 529 nm respectively.

### NGF withdrawal assay

Undifferentiated PC12 cells were plated onto collagen IV-coated plates (Corning) and differentiated in culture medium containing 50 ng/ml NGF (Accurate chemicals) for 5–7 days as described^20,21^. Differentiated PC12 were pre-treated with 10 mM N-acetylcysteine (NAC) (Sigma Aldrich) before NGF withdrawal and continuously replenished every 12 hours after NGF withdrawal with anti-NGF antibody (1:5000, Sigma Aldrich) for up to 48 hours where they were harvested for Immunoblot analysis.

### Proteasome activity assay

SK-N-SH cells were seeded at a density of 10,000 cells/100 µL/well in a 96-well plate. 100 mM diethyldithiocarbamate (DDC; Sigma Aldrich) were performed with PBS (Hyclone) and added into respective wells and incubated for 12 hours and 4 hours respectively at 37 °C, 5% CO_2_. A diluted solution of 8 µM Epoxomicin (ApexBio; Cat no. A2606) was added into respective well for 2 hours. Epoxomicin is an inhibitor control for this assay. Proteasome-Glo^TM^ Chymotrypsin-Like Cell-Based Assay Reagent (Promega) was performed according to manufacturer’s instructions. 100 µL/well of the proteasome reagent was added after equilibrated to room temperature. Cells containing the reagent were subjected to shaking at 600 rpm for 2 minutes, followed by 10 minutes incubation in the dark prior to luminescence measurement with a microplate reader. Luminescence was then measured and presented as RLU with mean ± S.D.

### Preparation and treatment of Gliotoxin

A stock solution of 1 mM Gliotoxin (ApexBio; Cat no. A4443) was prepared with DMSO and serial dilutions were prepared with PBS. Cells were seeded at 40% confluency in 60 mm culture dishes a day prior to treatment. To obtain the optimal time and doses, cells were treated with increasing doses of Gliotoxin at various time points.

### Crystal violet colony formation assay

Cells were seeded at 20% confluency in 60 mm culture dishes a day prior to Gliotoxin treatment. CHP212 and SK-N-SH cells were treated and replenished with 50 nM and 300 nM of Gliotoxin respectively for every 24 hours for 1 to 3 weeks.

### Flow cytometric analysis of apoptosis via PI/AV staining

Apoptotic activity was determined by the Dead Cell Apoptosis Kit with Annexin V FITC and PI (Molecular Probes, Thermo-Fisher Scientific; Cat no. V13241) according to manufacturer’s instructions. Cells were seeded at 70–80% confluency in 60 mm culture dishes a day prior to drug treatment. Cells were harvested in RPMI-1640 medium and cell pellets were washed with cold PBS before re-suspended with 100 µL of 1X Annexin-binding buffer. Cells were subsequently incubated with 2.5 µL of FITC Annexin V and 0.5 µL of 40 ng/mL PI working solution at room temperature for 15 minutes in the absence of light. Cell suspensions were further diluted and filtered before flow cytometric analysis using BD LSRFortessa^TM^ cell analyzer with excitation/emission spectra of 535 nm/617 nm for PI and 494 nm/518 nm for FITC respectively. At least 10,000 cells were analyzed and measured.

### Statistical analysis

Statistical analysis was performed by Student’s *t*-test unless stated otherwise. Statistically differences were reported as *p < 0.05, **p < 0.01, ***p < 0.001, ****p < 0.0001. All the figures shown represent result from at least three independent experiments.

### Data Availability Statement

All data generated or analyzed during this study are included in this published article (and its Supplementary Figures file).

## Electronic supplementary material

Supplementary Figures
