# Supplementary material for: KIF1Bβ increases ROS to mediate apoptosis and reinforces its protein expression through O2− in a positive feedback mechanism in neuroblastoma
Source: Sci Rep. 2017 Dec 4;7:16867. doi: 10.1038/s41598-017-17192-6 (PMC5715000; doi:10.1038/s41598-017-17192-6)

KIF1Bβ increases ROS to mediate apoptosis and reinforces its protein expression through O_2^- in a positive feedback mechanism in neuroblastoma

Clara Angelina[1#], Irene Sze Ying Tan[1#], Zhang'e Choo[1], Oswald Zhao Jian Lee[1], Shazib Pervaiz[1,2,3,4], Zhi Xiong Chen[1,5*]

[1]Department of Physiology, Yong Loo Lin School of Medicine, National University of Singapore, Singapore (I.S.Y.T., C.A., O.Z.J.L., Z.C., S.P., Z.X.C.)

[2]NUS Graduate School for Integrative Sciences and Engineering, National University of Singapore, Singapore (S.P.)

[3]Cancer and Stem Cell Biology Program, Duke-NUS Graduate Medical School, Singapore (S.P.)

[4]Singapore-MIT Alliance, Singapore (S.P.)

[5]KK Women's and Children's Hospital, Singapore (Z.X.C.)

[#]Both authors contributed equally

*Corresponding author:

Dr. Zhi Xiong Chen

Department of Physiology, Yong Loo Lin School of Medicine, National University of Singapore, MD9, 2 Medical Drive, S117597

Tel: (+65) 6516 3231 / Fax: (+65) 6778 8161 / Email: zhixiong_chen@nus.edu.sg

Supplementary Figure Legends

Figure S1: Overexpression of wild-type KIF1Bβ and KIF1Bβ600-1400 has no effect on mitochondrial O_2^- in NB1 cells.

(A) Immunoblot analysis of NB1 cells transfected with empty vector pcDNA3 (Empty), wild-type KIF1Bβ (FL) and KIF1Bβ600-1400 (600-1400). **(B)** Corresponding mitochondrial O_2^- measurement conducted using MitoSOX Red assay after 24 hours of transfection.

Figure S2: O_2^- increases endogenous KIF1Bβ protein expression.

(A) Immunoblot analysis of CHP212 and **(B)** NB1 cells transfected with empty vector pcDNA3 (Empty), wild-type KIF1Bβ (FL) or wild-type KIF1Bβ treated with NAC for 48 hours (Cells were pre-incubated with NAC for 2 hours). Right – corresponding densitometry for FLAG-KIF1Bβ expression. **(C)** Immunoblot analysis of endogenous KIF1Bβ expression in SK-N-SH cells upon treatment with DPI or DDC alone for 4 hours, or in combination (pre-incubate DPI for 12 hours, followed by 4 hours treatment of DDC). Right – densitometry of KIF1Bβ expression. **(D)** Corresponding intracellular O_2^- measurement determined using lucigenin assay in SK-N-SH cells treated with DPI or DDC alone or in combination (mean ± SD; n=3; *, $P<0.05$; ***, $P<0.001$). **(E)** Intracellular O_2^- measurement determined using lucigenin-based chemiluminescence assay after 4 hours of treating SK-N-SH cells with increasing doses of DDC.

Figure S3: Dose- and time-dependent response of KIF1Bβ expression upon treatment with Gliotoxin in neuroblastoma cell lines.

(A) Immunoblot analysis of CHP212 and **(B)** SK-N-SH cells in response to increasing doses of Gliotoxin treatment for 24 hours. **(C)** Immunoblot analysis of CHP212 cells treated with 50 nM Gliotoxin and **(D)** SK-N-SH cells treated with 300 nM Gliotoxin over 72 hours. **(E)** Crystal violet staining to determine colony formation ability of CHP212 and **(F)** SK-N-SH cells that were treated with Gliotoxin for every 24 hours (50 nM) and 12 hours (300 nM) respectively, for several days. **(G)** Immunoblot analysis of endogenous KIF1Bβ expression in CHP212 cells and **(H)** SK-N-SH cells treated with 50 nM or 300 nM Gliotoxin, respectively for 24 hours, or in combination with 10mM Tiron for 24 hours (Cells were pre-treated with 10 mM Tiron for 2 hours). Bottom – densitometry of KIF1Bβ expression.

Figure S4: Overexpression of EglN3 increases mitochondrial O_2^- in SK-N-SH cells.

(A) Immunoblot analysis of FLAG-EglN3 expression after 24 hours of transfection in SK-N-SH cells. **(B)** Corresponding total ROS measurement determined using DCFDA assay 24 hours post-transfection with 1 μg or 2 μg FLAG-EglN3 plasmids. **(C)** Corresponding mitochondrial O_2^- measurement determined using MitoSOX Red assay after 24 hours of transfection with 1 μg or 2 μg FLAG-EglN3 (mean ± SD; n=3; **, P<0.01).

Supplementary Figure 1

A

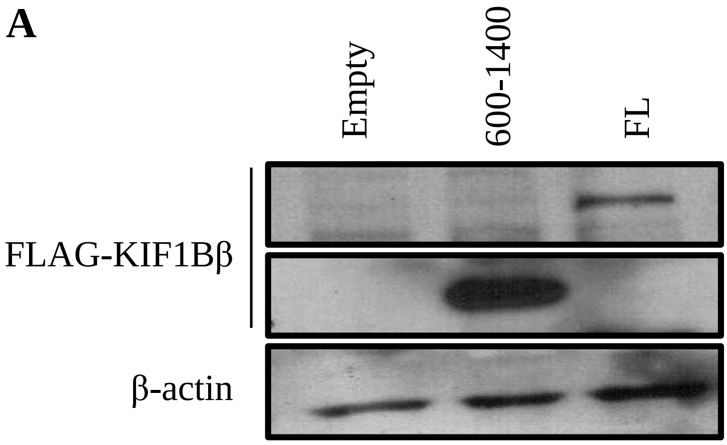

B

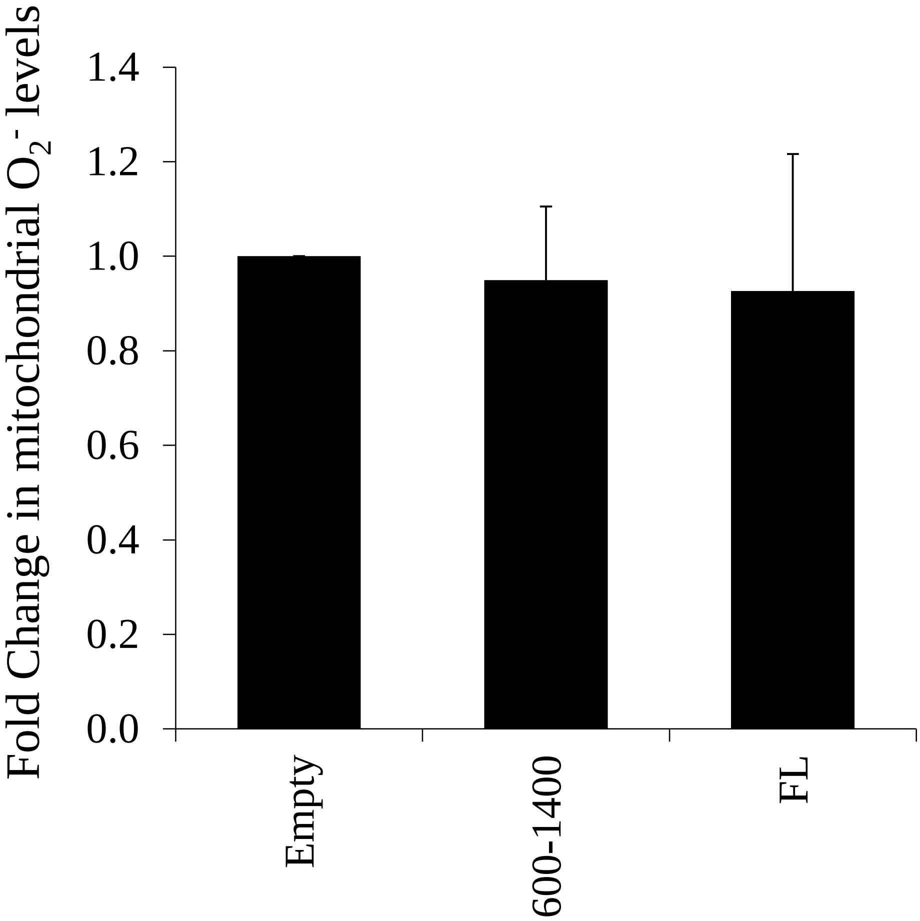

Supplementary Figure 2

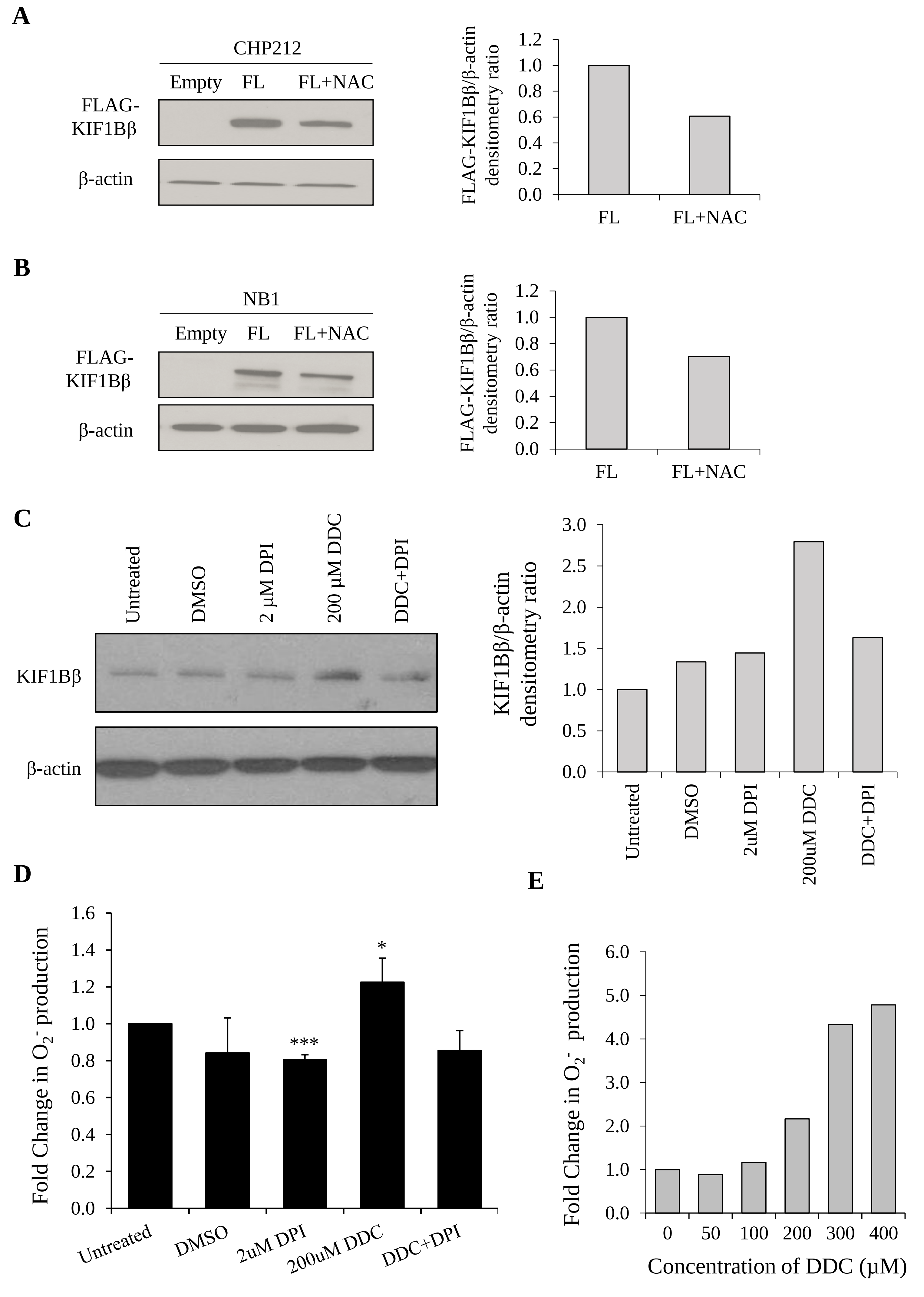

Supplementary Figure 3

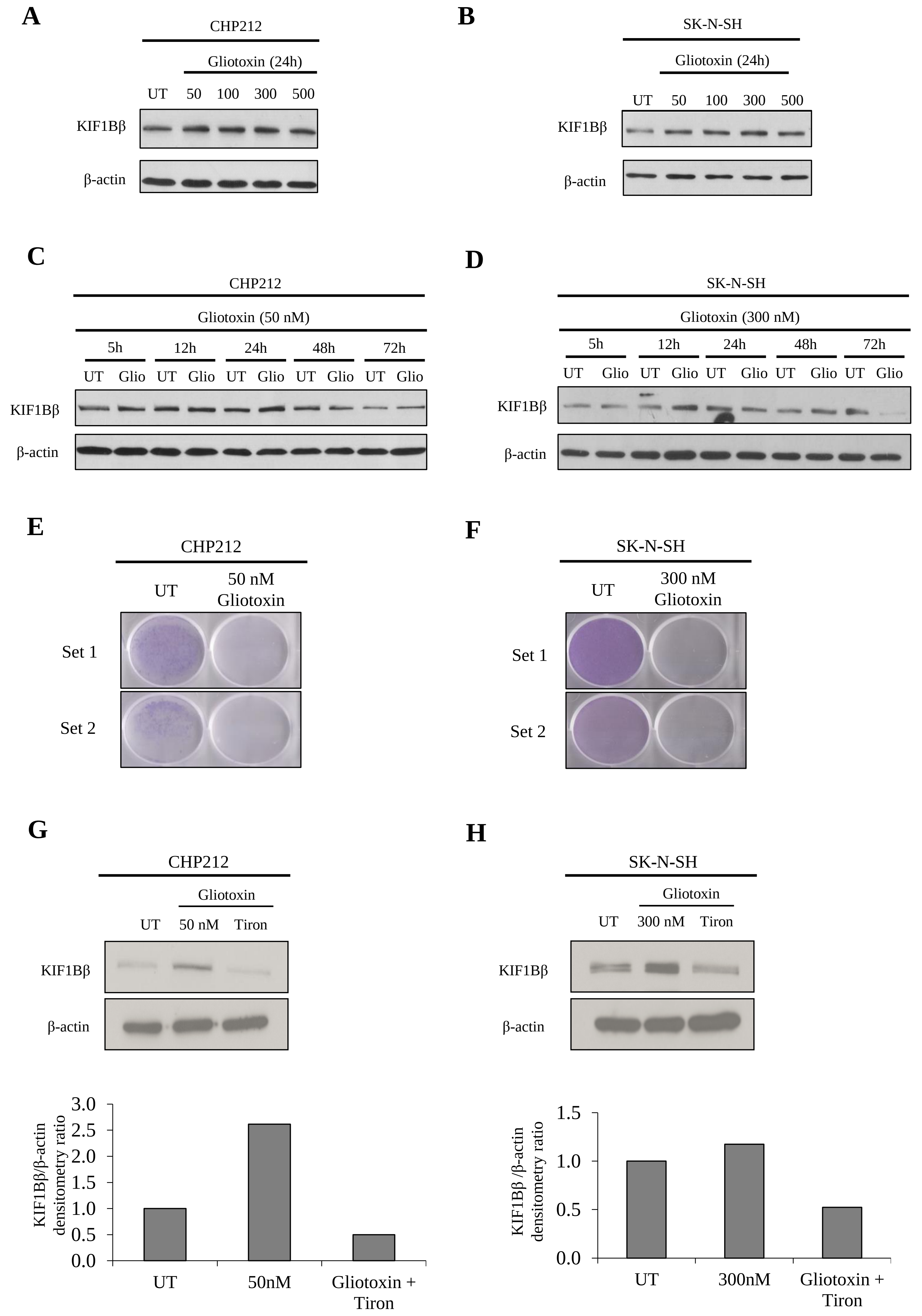

Supplementary Figure 4

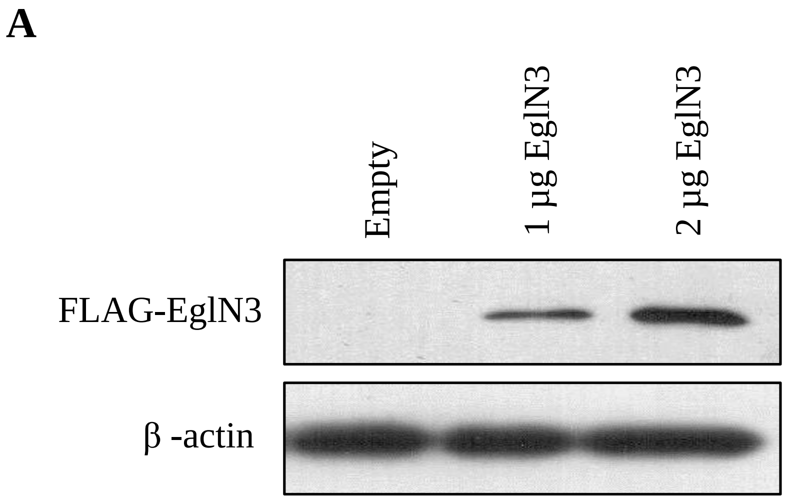

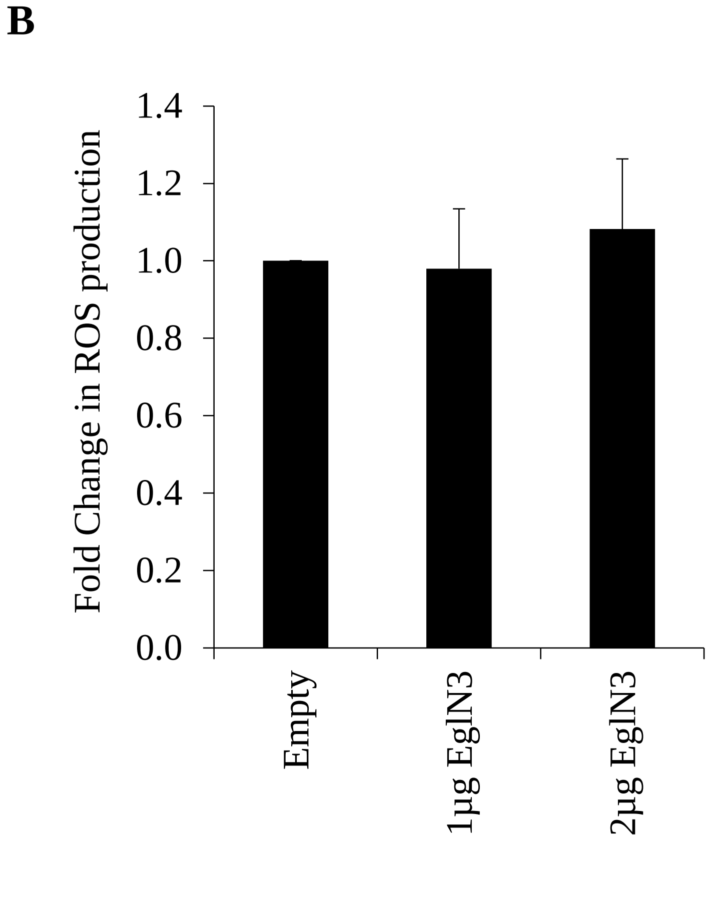

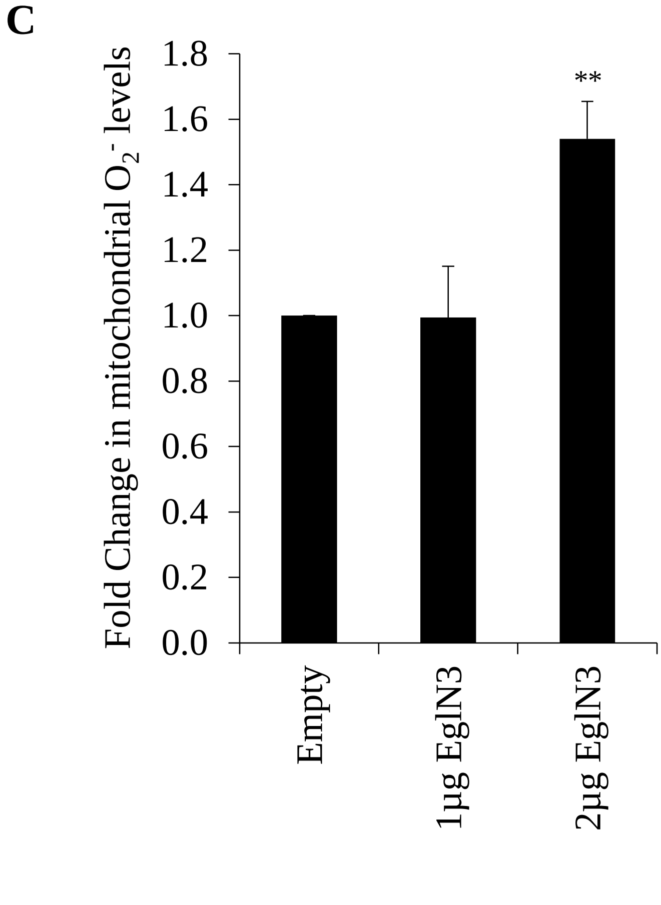

Supplement: Supplementary file 1 — Supplementary Figures [file 41598_2017_17192_MOESM1_ESM.pdf]
